# Correlation Between Cognitive Function and Serum Irisin Levels in Parkinson's Disease Patients

**DOI:** 10.1002/brb3.71848

**Published:** 2026-10-21

**Authors:** Meilin Gui, Lixia Qin, Lingling Lv, Jinduo Huang, Shenglan Hu, Kexin Chen, Xing Wang, Yiwen Zhou, Beisha Tang, Hainan Zhang, Chunyu Wang

**Affiliations:** ^1^ Department of Neurology, Second Xiangya Hospital Central South University Changsha China; ^2^ Clinical Medical Research Center for Stroke Prevention and Treatment of Hunan Province, Department of Neurology, Second Xiangya Hospital Central South University Changsha China; ^3^ Key Laboratory of Hunan Province in Neurodegenerative Disorders Central South University Changsha China; ^4^ Center For Medical Genetics, School of Life Sciences Central South University Changsha China; ^5^ National Clinical Research Center for Geriatric Disorders Xiangya Hospital Changsha China; ^6^ Department of Neurology, Xiangya Hospital Central South University Changsha China; ^7^ Department of Medical Genetics The Second Xiangya Hospital Central South University Changsha China; ^8^ Department of Medical Genetics, Hunan Province Clinical Medical Research Center For Genetic Birth Defects and Rare Diseases The Second Xiangya Hospital of Central South University Changsha Hunan Province China

**Keywords:** Parkinson's disease, cognitive impairment, irisin, motor subtypes

## Abstract

**Introduction:**

Cognitive impairment is a common non‐motor symptom of Parkinson's disease (PD) that markedly reduces patients’ quality of life. Given the clinical heterogeneity of PD‐related cognitive impairment (PDCI), further clarification of clinical and circulating biological factors associated with cognitive status may help improve our understanding of cognitive dysfunction in PD.

**Methods:**

In this cross‐sectional study, 243 patients with PD and 90 age‐ and sex‐matched healthy controls (HCs) were enrolled. Cognitive function was evaluated using the Montreal Cognitive Assessment (MoCA), and patients with PD were classified into the PD with cognitive impairment (PDCI) and PD without cognitive impairment (PDNCI) groups. Based on motor symptoms, PD patients were further divided into three subtypes: tremor‐dominant (TD), postural instability and gait disorder (PIGD), and indeterminate subtype (IND). Group comparisons and logistic regression analyses were performed to examine factors associated with cognitive impairment.

**Results:**

Serum irisin levels did not differ significantly between patients with PD and healthy controls (*p =* 0.478). Compared with PDNCI patients, those with PDCI had lower serum irisin levels. Among motor subtypes, the PIGD group showed a higher prevalence of cognitive impairment (68.8% vs. 46.2%, *p <* 0.001) and significantly lower irisin levels compared with the TD group [3.25 (2.62–4.14) vs. 4.12 (3.42–5.07) µg/mL, *p <* 0.001]. Serum irisin levels were weakly but positively associated with memory performance, and this association remained significant after FDR correction across the five cognitive domains (*r =* 0.194, FDR‐adjusted *p =* 0.010). Compared with patients in the TD–high irisin group, those in the PIGD–low irisin group had higher odds of concurrent cognitive impairment (OR = 6.065, 95% CI: 2.974–18.638, *p =* 0.002).

**Conclusion:**

Cognitive impairment was common among patients with PD, particularly those with the PIGD subtype. The PIGD subtype and lower serum irisin levels were associated with the presence of PDCI. In the joint‐category analysis, patients in the PIGD‐low‐irisin group had higher odds of concurrent cognitive impairment than those in the TD‐high‐irisin group.

## Graphical abstract

In this cross‐sectional study, the PIGD subtype and lower serum irisin levels were associated with cognitive impairment in patients with Parkinson’s disease. Patients in the PIGD–low‐irisin group had higher odds of concurrent cognitive impairment than those in the TD–high‐irisin group.

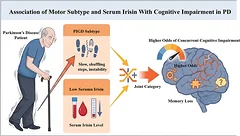

## 1 Introduction

Parkinson's disease (PD) is the second most prevalent neurodegenerative disorder, characterized by the progressive degeneration of nigrostriatal dopaminergic neurons and the widespread intracellular aggregation of α‐synuclein (α‐Syn) (Karikari et al. 2018). Clinically, PD is defined by its hallmark motor symptoms—bradykinesia, resting tremor, muscular rigidity, and postural or gait disturbances. However, a broad spectrum of non‐motor symptoms, including sleep disorders, autonomic dysfunction, and cognitive impairment, also occurs frequently. Among these, cognitive impairment represents a particularly burdensome feature, substantially diminishing patients’ quality of life. Its onset and progression vary widely among individuals, spanning a continuum from normal cognition to mild cognitive impairment (MCI) and, ultimately, PD dementia (PDD) (Aarsland et al. 2021). Epidemiological studies indicate that approximately one‐third of PD patients present with mild cognitive impairment at diagnosis (Muslimović et al. 2005, Broeders et al. 2013). Within five years, up to half of patients develop MCI, and after 15–20 years, as many as 80% may progress to PDD (Hely et al. 2008). Cognitive decline not only compromises patients’ functional independence but also contributes to greater disability and care dependence. Early identification and intervention are therefore critical for improving clinical outcomes. Accordingly, investigating clinical phenotypes and circulating biological factors associated with PD‐related cognitive impairment (PDCI) may help improve our understanding of cognitive heterogeneity in PD.

The pathogenesis of cognitive impairment in PD has not yet been fully elucidated and is likely influenced by multiple interacting factors, including aging, disease severity, motor phenotype, physical activity, and metabolic status. Ageing is one of the most important determinants of cognitive decline in PD and may also affect biological pathways linking motor dysfunction, systemic metabolism, and cognition. With advancing age and disease progression, patients with PD often experience reduced skeletal muscle function, decreased physical activity, and metabolic dysregulation, all of which may contribute to cognitive vulnerability. Motor subtype may further modify this relationship. Previous studies have reported that patients with the postural instability–gait disorder (PIGD) subtype tend to have a higher prevalence of cognitive impairment than those with other motor phenotypes (Jankovic et al. 1990; Alves et al. 2006; van der Heeden et al. 2016). Because PIGD patients commonly present with gait and balance impairment, they may experience greater mobility restriction and reduced muscle activity. These clinical features suggest that muscle‐derived metabolic mediators may provide a biologically relevant link among aging, motor phenotype, physical activity, and cognitive impairment in PD

Among these muscle‐derived mediators, irisin has attracted increasing attention as a potential link between skeletal muscle activity, metabolic regulation, and brain health. Irisin, a myokine first identified by Boström et al. in 2012, is a cleavage product of the fibronectin type III domain‐containing protein 5 (FNDC5), which is induced by the overexpression of peroxisome proliferator‐activated receptor gamma coactivator‐1α (PGC‐1α) (Boström et al. 2012). Secreted primarily by skeletal muscle, irisin circulates in the bloodstream and exerts multiple systemic effects (Kim et al. 2018). Initial research focused on its role in metabolic regulation, demonstrating that irisin promotes the browning of white adipose tissue, enhances energy expenditure, improves glucose and lipid metabolism, and increases insulin sensitivity—thereby linking it closely to metabolic disorders such as obesity and type 2 diabetes (Hee Park et al. 2013; Boström and Fernández‐Real 2014).

Notably, irisin has attracted attention in neurodegenerative disorders and brain health. Some studies have reported that factors such as irisin have a neuroprotective effect and can improve the functioning of the nervous system after central nervous system injury (Dun et al. 2013; Lourenco et al. 2019). One proposed pathway involves the PGC‐1α/FNDC5/irisin axis and downstream regulation of brain‐derived neurotrophic factor (BDNF), which is involved in hippocampal synaptic plasticity, long‐term potentiation, learning, and memory (Lourenco et al. 2019; Kowiański et al. 2018; Wrann et al. 2013). However, BDNF represents only one potential mechanism, and the relevance of circulating irisin in PD is likely multifactorial. In PD, aging, disease severity, and motor phenotype may influence muscle activity, metabolic status, and neurodegenerative vulnerability.

More recently, some studies have implicated irisin as having been observed to prevent the loss of dopamine neurons and the reduction of striatal dopamine levels and to improve motor dysfunction in PD patients (Kam et al. 2022; Zhang et al. 2022). Existing clinical evidence has also begun to link irisin with PD severity and cognitive function. For example, Shi et al. reported that irisin levels in the plasma of PD patients were negatively correlated with the severity of motor symptoms and cognitive impairment (Shi et al. 2024).

Despite growing interest, to date there have been very few studies examining the relationship between irisin levels and cognitive impairment across the different subtypes of PD. Therefore, the present study aimed to investigate serum irisin in relation to cognitive function and motor subtype. Furthermore, we investigated the associations between serum irisin concentrations and specific cognitive domains, with the goal of providing novel insights and empirical evidence to support the early identification and management of cognitive impairment in PD.

## 2 Methods

### 2.1 Study Participants

This cross‐sectional study included 243 patients diagnosed with PD and 90 age‐ and sex‐matched healthy controls (HCs), consecutively enrolled at Second Xiangya Hospital of Central South University from September 2022 to February 2025. The diagnosis of PD was established according to the United Kingdom Parkinson's Disease Brain Bank criteria (Hughes et al. 1992). Eligible participants were 45–80 years of age and had no history of dementia, major psychiatric disorders, other neurodegenerative diseases, or prior functional neurosurgery for PD. All diagnoses were independently confirmed by two experienced movement disorder neurologists.

HCs were recruited from spouses or siblings of PD patients and community volunteers from the same geographic region. They were matched to the PD cohort by age and sex. Exclusion criteria for both PD and HC groups included acute or chronic infections, hematological disorders, autoimmune or rheumatic diseases, thyroid dysfunction (hypo‐ or hyperthyroidism), malignancy, severe hepatic or renal dysfunction, significant visual or auditory impairment, and communication difficulties.

All participants underwent face‐to‐face evaluations conducted by trained investigators who had passed inter‐rater reliability testing. The study protocol was approved by the Ethics Committee of the Second Xiangya Hospital, Central South University (Approval No. LYEC2025‐0227), and written informed consent was obtained from all participants.

### 2.2 Measurements

Demographic and clinical data, including age, sex, disease duration, education level, and comorbidities, were collected through structured interviews and medical records. Hypertension was defined as a systolic blood pressure ≥140 mmHg and/or a diastolic blood pressure ≥90 mmHg, a previous physician diagnosis of hypertension, or current use of antihypertensive medication. Fasting venous blood samples were collected by trained nurses in the early morning after 6–8 h of fasting. Laboratory assessments included hemoglobin, albumin, glomerular filtration rate (GFR), calcium, phosphorus, triglycerides, total cholesterol, high‐density lipoprotein (HDL), low‐density lipoprotein (LDL), and fasting glucose. Serum irisin concentrations were quantified using a commercial Human Irisin ELISA Kit (AF1912‐A, Hunan AiFang Biological, Beijing, China). Serum samples were diluted fivefold before measurement, and the reported concentrations were corrected using this dilution factor. The standard‐curve range for the kit lot used in this study was 0–5.0 µg/mL, with a sensitivity of 1.0 ng/mL, and the intra‐ and interassay variability was < 15 %.

Disease severity was classified according to the Hoehn and Yahr (H–Y) staging scale: stages 1–2 were defined as early, stages 2.5–3 as intermediate, and stages 4–5 as advanced (Goetz et al. 2004). Motor symptoms were evaluated using the Unified Parkinson's Disease Rating Scale Part III (UPDRS‐III) in the medication OFF state (discontinue dopaminergic medications for at least 12 h), which consists of 18 sections and 33 items. Each item is scored based on symptom severity, yielding a total score ranging from 0 to 132, with higher scores indicating greater motor impairment. Motor subtype classification was performed according to the classic tremor‐dominant/postural instability–gait disorder classification scheme (Jankovic et al. 1990). Patients were categorized as tremor‐dominant (TD, mean TD/PIGD ratio ≥ 1.5), postural instability–gait disorder (PIGD, mean TD/PIGD ratio ≤ 1.0), and indeterminate (IND, 1.0< TD/PIGD ratio < 1.5). The IND group was excluded from the motor‐subtype‐irisin joint‐category analysis because it did not meet the criteria for a clearly predominant TD or PIGD phenotype. All assessments were performed independently by two trained clinicians, and the mean of their scores was used for analysis.

### 2.3 Cognitive Function Assessment

Cognitive assessments were administered by two trained neuropsychologists, and the averaged results were used for analysis. The Montreal Cognitive Assessment (MoCA) was selected due to its superior sensitivity compared with the Mini‐Mental State Examination (MMSE) in detecting early cognitive decline and its reduced ceiling and floor effects (Snyder et al. 2021; Weintraub et al. 2004). The MoCA evaluates seven cognitive domains with a maximum score of 30, where lower scores indicate poorer cognition. Participants with fewer than 12 years of education received one additional point, and a total score <26 was defined as cognitive impairment (Dalrymple‐Alford et al. 2010). For the exploratory cognitive‐severity analysis, patients with PDCI were further stratified according to their education‐adjusted MoCA scores into a milder cognitive‐impairment group (MoCA 21–25) and a more severe cognitive‐impairment group (MoCA <21). These MoCA‐based categories were used to characterize the severity of cognitive screening scores and were not considered equivalent to formal diagnoses of PD‐MCI or PDD.

Following the classification proposed by Lam et al. (2013), five cognitive domains were further analyzed: ① Memory–digit span forward, delayed recall, and orientation (12 points); ② Visuospatial ability—cube copying and clock drawing (4 points); ③ Verbal ability—animal naming and sentence repetition (5 points); ④ Attention serial 7 subtraction and digit span backward (4 points); ⑤ Executive function—trail making, digit reversal, word association, and verbal fluency (5 points).

### 2.4 Statistical Analysis

All statistical analyses were conducted using SPSS version 27.0 (IBM Corp., Armonk, NY, USA) and GraphPad Prism version 8.0 (GraphPad Software, San Diego, CA, USA). Data normality was evaluated using the Shapiro–Wilk test. Normally distributed variables are presented as mean ± standard error (SE), whereas non‐normally distributed variables are expressed as median (interquartile range, IQR). Between‐group comparisons were performed using Student's t‐test (parametric), Mann–Whitney U test (non‐parametric), or χ^2^ test (categorical variables).

Univariable binary logistic regression analyses were performed to examine factors associated with the presence of PDCI, followed by multivariable analysis to adjust for potential confounders. For the domain‐specific cognitive correlation analyses, partial correlation was used to adjust for potential confounders, including age and years of education. To account for multiple comparisons across the five cognitive domains, false discovery rate (FDR) correction was applied to the corresponding P values. A two‐tailed p‐value <0.05 was considered statistically significant.

## 3 Results

### 3.1 Demographic and Clinical Characteristics of Participants

A total of 243 patients with PD (mean age, 61.63 ± 10.38 years) and 90 HC (mean age, 59.67 ± 10.08 years) were included in this study. The prevalence of cognitive impairment among PD patients was 54.3%, significantly higher than that in the HC group (17.8%, *p <* 0.05). No significant differences were observed between the two groups in terms of demographic characteristics (sex, body mass index [BMI], education, hypertension, or diabetes) or laboratory indices such as homocysteine and serum irisin levels (*p* > 0.05). Detailed statistics are presented in Table 1.

**TABLE 1:** Comparison of general demographic and clinical data between the PD group and the HC group.

Feature	PD (*n =* 243)	HC (90)	Z/t/χ2	*p*‐value
Sex female (n, %)	(113, 46.5%)	(47, 52.2%)	0.861	0.353
Age (years)	61.63 ± 10.38	59.67 ± 10.08	1.544	0.123
BMI (Kg/m^2^)	23.59 ± 3.29	24.21 ± 3.00	− 1.567	0.118
Level of education (years)	8.0(5.0,9.0)	7.0(6.0,9.0)	− 1.309	0.191
Hypertension (n, %)	(65, 26.7%)	(20, 22.2%)	0.708	0.400
Diabetes mellitus (n, %)	(29, 11.9%)	(9, 10.0%)	0.243	0.622
Hemoglobin (g/L)	133.5 (123.0,143.0)	134.0(124.0,145.0)	− 0.756	0.449
Total protein (g/L)	68.26 ± 5.88	69.37 ± 6.64	− 1.467	0.143
Albumin (g/L)	41.10 (38.50,44.95)	37.90(36.20,42.40)	− 0.125	0.901
Triglyceride (mmol/L)	1.20 (0.89,1.72)	1.71(1.27,2.25)	− 4.068	**<0.001**
Total cholesterol (mmol/L)	4.46 (3.85,5.13)	4.49 ± 0.73	− 0.273	0.785
HDL (mmol/L)	1.25 (1.03,1.50)	1.13(0.96,1.44)	− 2.467	**0.014**
LDL (mmol/L)	2.75 (2.04,3.28)	2.60(2.23,2.89)	− 0.518	0.605
Calcium (mmol/l)	2.24 (2.16,2.31)	2.21(2.16,2.26)	− 1.288	0.199
Phosphorus (mmol/l)	1.11 (0.93,1.25)	1.12(0.98,1.24)	− 0.704	0.482
GFR (ml/min/1.73m^2^)	84.66 (66.39,95.74)	80.50(60.71,95.43)	− 0.684	0.494
Homocysteine (µmol/L)	12.00 (9.82,14.13)	11.80(10.80,15.50)	− 0.769	0.442
Fasting Blood‐glucose (mmol//L)	4.98 (4.44,5.61)	4.68(4.11,5.31)	− 0.126	0.900
Irisin (µg/mL)	3.87 (3.10,4.68)	3.52 (3.09,4.83)	− 0.709	0.478
Cognitive impairment (n, %)	(132, 54.3%)	(16, 17.8%)	35.520	**<0.001**

Abbreviations: BMI: body mass index; GFR: glomerular filtration rate; HC: healthy control; HDL: high‐density lipoprotein; LDL: low‐density lipoprotein; PD: Parkinson's disease.

Based on cognitive status, PD patients were categorized into the PDCI group (*n =* 132, 54.3%) and the PDNCI group (*n =* 111, 45.7%). Compared with the PDNCI group, patients with PDCI were older (65.30 ± 9.37 vs. 57.27 ± 9.86 years, *p <* 0.001), had a longer disease duration, higher H–Y stage, higher UPDRS‐III scores, and elevated homocysteine levels. Conversely, the PDCI group had lower educational attainment [7.00 (5.00–9.00) vs. 8.00 (7.00–9.00), *p <* 0.001], lower albumin concentrations (41.02 ± 4.02 vs. 42.34 ± 4.30 g/L, *p =* 0.014), and reduced serum irisin levels [3.52 (2.93–4.60) vs. 4.07 (3.31–4.82) µg/mL, *p =* 0.013] (Figure 1A). No significant differences were noted in sex distribution, comorbidities (hypertension, diabetes), or other laboratory parameters (lipid profile, GFR, 25‐hydroxyvitamin D; *p* > 0.05). Details are shown in Table 2.

**FIGURE 1:**
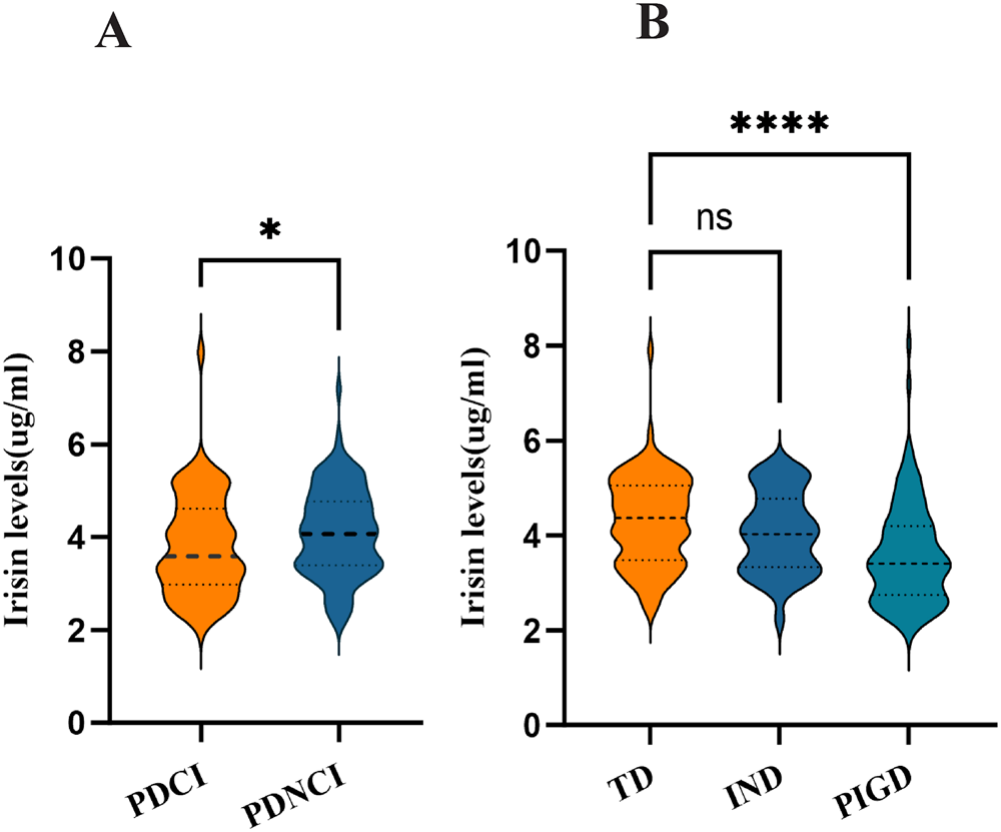
**(A)** Comparison of serum irisin levels between PDCI and PDNCI groups. (B) Comparison of serum irisin levels between TD, IND and PIGD groups. PDCI: Parkinson's disease with cognitive impairment, PDNCI: Parkinson's disease without cognitive impairment, TD: tremor‐dominant, PIGD: postural instability‐ gait disorder. *: *p <* 0.05, **: *p <* 0.01, ***: *p <* 0.001.

**TABLE 2:** Comparison of general demographic and clinical data between the PDCI group and the PDNCI group.

Feature	PDCI (*n =* 132)	PDNCI (*n =* 111)	z/t/χ2	*p* value
Sex, female (n, %)	(68, 51.5%)	(45, 40.5%)	2.919	0.065
Age (years)	65.30 ± 9.37	57.27 ± 9.86	− 6.494	< 0.001
BMI (Kg/m^2^)	23.44 ± 3.21	23.74 ± 3.20	0.648	0.517
Level of education (years)	7.0 (5.0,9.0)	8.0 (7.0,9.0)	− 4.914	< 0.001
Hypertension (n, %)	37 (28.0%)	28 (25.2%)	0.242	0.678
Diabetes mellitus (n, %)	19 (14.4%)	10 (9.0%)	1.664	0.214
Duration of illness (years)	4.0 (2.0,8.0)	2.0 (1.0,3.0)	−5.633	< 0.001
H‐Y stage	2.5 (2.0,3.0)	1.5 (1.0,2.5)	−7.172	< 0.001
UPDRS III (score)	38.0 (29.0,48.0)	23.0 (17.0,29.0)	−7.826	< 0.001
Hemoglobin (g/L)	132.0 (120.5,141.5)	135.0 (124.0,146.0)	−1.730	0.084
Total protein(g/L)	68.29 ± 5.83	68.24 ± 5.96	−0.065	0.948
Albumin (g/L)	41.02 ± 4.02	42.34 ± 4.30	2.468	0.014
Triglyceride (mmol/L)	1.20 (0.87,1.79)	1.21 (0.92,1.63)	−0.482	0.630
Total cholesterol (mmol/L)	4.55 (3.86,5.25)	4.32 (3.83,5.07)	−0.298	0.766
HDL (mmol/L)	1.26 (1.01,1.55)	1.24 (1.07,1.47)	−0.542	0.588
LDL (mmol/L)	2.74 (2.05,3.41)	2.77 (2.04,3.19)	−0.118	0.906
Calcium (mmol/l)	2.25 (2.17,2.31)	2.22 (2.15,2.32)	−1.092	0.275
Phosphorus (mmol/l)	1.13 (0.94,1.26)	1.10 (0.93,1.26)	−0.919	0.358
GFR (ml/min/1.73m^2^)	82.44 (64.52,92.91)	85.54 (72.96,99.06)	−1.818	0.069
25‐hydroxyvitamin D(nmol/L)	48.90 ± 15.27	52.34 ± 17.50	1.631	0.104
Homocysteine (µmol/L)	12.40 (9.90,15.00)	11.70 (9.80,13.45)	−2.161	**0.031**
CRP (mg/L)	1.80 (1.00,3.64)	1.64 (0.76,2.32)	−1.270	0.204
Fasting Blood‐glucose(mmol//L)	4.92 (4.43,5.66)	5.08 (4.47,5.52)	−0.025	0.980
Irisin (µg/mL)	3.52 (2.93,4.60)	4.07 (3.31,4.82)	−2.489	**0.013**

Abbreviations: BMI: body mass index; CRP: C‐reactive protein; GFR: glomerular filtration rate; H‐Y: Hoehn‐Yahr; HDL: high‐density lipoprotein; LDL: low‐density lipoprotein; PDCI: Parkinson's disease with cognitive impairment; PDNCI: Parkinson's disease without cognitive impairment.

Furthermore, PD patients were subclassified by motor phenotype into TD (*n =* 93, 38.3%), IND (*n =* 54, 22.2%), and PIGD (*n =* 96, 39.5%) subtypes. The prevalence of cognitive impairment was significantly higher in the PIGD group compared with the TD and IND group (68.8% vs. 46.2% vs. 42.6%, *p <* 0.001). Patients in the PIGD subtype also exhibited longer disease duration [3.0 (2.0–7.0) vs. 2.0 (1.0–5.0) vs. 3.00(1.50–7.00) years, *p =* 0.019], higher H–Y stage [2.5 (1.5–3.0) vs. 1.00(1.00,2.00) vs. 1.5 (1.0–3.0), *p =* 0.049], and lower serum irisin concentrations [3.25 (2.62–4.14) vs. 3.93(3.41‐4.92) vs. 4.12 (3.42–5.07) µg/mL, *p <* 0.001] compared with the TD and IND group (Figure 1B). No significant differences were observed between groups in sex, age, or educational attainment (*p* > 0.05). Comprehensive data are provided in Table 3.

**TABLE 3:** Comparison of serum irisin levels and proportion of cognitive impairment subtypes among different motor subtypes of PD.

Feature	TD(*n =* 93)	IND (54)	PIGD(*n =* 96)	F/H/χ2	*p* value
Sex female(n, %)	(42, 45.2%)	(24, 44.4%)	(47, 49.0%)	0.392	0.822
Age (years)	60.26 ± 10.92	60.74 ± 9.49	63.46 ± 10.16	2.531	0.082
BMI(Kg/m^2^)	24.21 ± 3.03	23.50 ± 3.68	23.05 ± 3.24	3.048	0.051
Level of education (years)	7.00 (6.00,9.00)	8.00 (6.00,9.00)	8.00 (5.00,9.00)	0.205	0.902
Duration of illness (years)	2.00 (1.00,5.00)^a^	3.00 (1.50,7.00)^ab^	3.00 (2.00,7.00)^b^	7.935	**0.019**
H‐Y stage	1.00 (1.00,2.00)	1.50 (1.00,3.00)	2.50 (1.50,3.00)	3.117	**0.049**
UPDRS III(score)	28.0 (19.0,40.0)	29.0 (19.0,40.0)	30.0 (22.5,45.0)	2.591	0.274
Hemoglobin(g/L)	136.0 (124.0,148.0)	130.0 (123.0,140.0)	132.0 (121.0,143.5)	6.281	**0.043**
Total protein(g/L)	70.55 (64.58,74.05)	66.40 (64.90,70.70)	67.50 (64.35,72.20)	3.479	0.176
Albumin (g/L)	42.25 (38.58,45.93)	40.60 (38.50,43.00)	40.80 (38.25,44.50)	5.142	0.076
Triglyceride (mmol/L)	1.20 (0.93,1.71)	1.39 (0.92,1.71)	1.21 (0.83,1.79)	0.289	0.866
Total cholesterol (mmol/L)	4.51 (3.85,5.13)	4.30 (3.85,5.28)	4.54 (3.81,5.04)	0.259	0.878
HDL(mmol/L)	1.25 (1.01,1.47)	1.21 (1.06,1.43)	1.29 (1.05,1.61)	0.076	0.963
LDL(mmol/L)	2.85 (2.25,3.39)	2.61 (2.01,3.43)	2.71 (2.03,3.18)	1.319	0.517
Calcium (mmol/l)	2.26 (2.15,2.31)	2.22 (2.16,2.31)	2.24 (2.16,2.33)	1.811	0.404
Phosphorus (mmol/l)	1.10 (0.90,1.28)	1.14 (0.93,1.25)	1.11 (0.96,1.24)	0.305	0.859
GFR (ml/min/1.73m^2^)	85.29 (61.89,96.91)	84.69 (69.00,92.90)	84.62 (72.70,96.77)	0.149	0.928
25‐hydroxyvitamin D(nmol/L)	47.50 (37.00,58.00)	46.00 (33.00,65.00)	48.00 (38.50,58.50)	0.119	0.942
Homocysteine (umol/L)	11.60 (9.70,14.05)	12.00 (9.70,14.00)	12.10 (9.90,14.39)	0.554	0.758
CRP(mg/L)	1.62 (0.77,2.65)	1.70 (0.87,3.60)	1.75 (1.03,2.93)	0.565	0.754
Fasting Blood‐glucose(mmol//L)	5.25 (4.66,5.81)^a^	4.68 (4.23,5.20)^b^	4.82 (4.45,5.41)^b^	9.759	0.008
Irisin (µg/mL)	4.12 (3.42,5.07)^a^	3.93 (3.41,4.92)^a^	3.25 (2.62,4.14)^b^	34.782	< 0.001
MMSE (score)	26.00 (23.00,28.00)^a^	27.00 (25.00,29.00)^a^	24.00 (21.50,26.50)^b^	22.048	< 0.001
MoCA (score)	24.50 (18.00,25.00)^a^	25.00 (18.00,25.00)^a^	19.00 (15.50,25.00)^b^	14.444	< 0.001
Cognitive impairment (n, %)	(43, 46.2%)^a^	(23, 42.6%)^a^	(66, 68.8%)^b^	13.498	< 0.001

Note: The *p* value represents the overall comparison among the three groups. Values sharing the same superscript letter are not significantly different, whereas values with no common letter differ significantly after post hoc correction, adjusted *p* < 0.05.

Abbreviations: BMI: body mass index; CRP: C‐reactive protein; GFR: glomerular filtration rate; H‐Y: Hoehn‐Yahr; HDL: high‐density lipoprotein; IND: indeterminate; LDL: low‐density lipoprotein; PIGD: postural instability and gait disorder; TD: tremor‐dominant.

### 3.2 Factors Associated with PDCI

Stepwise logistic regression analysis was performed based on the comparative results in Table 2. In univariate analyses, old age (OR = 1.090, 95% CI 1.058–1.124, *p <* 0.001), longer disease duration (OR = 1.281, 95% CI 1.159–1.416, *p <* 0.001), higher H–Y stage (OR = 3.745, 95% CI 2.507–5.593, *p <* 0.001), higher UPDRS‐III score (OR = 1.096, 95% CI 1.067–1.125, *p <* 0.001), and higher homocysteine levels (OR = 1.096, 95% CI 1.018–1.180, *p =* 0.016) were associated with higher odds of having PDCI. Conversely, more years of education (OR = 0.813, 95% CI 0.744–0.888, *p <* 0.001), higher serum albumin levels (OR = 0.926, 95% CI 0.870–0.985, *p =* 0.015), and higher serum irisin levels (OR = 0.764, 95% CI 0.594–0.983, *p =* 0.036) were associated with lower odds of having PDCI.

In the multivariable binary logistic regression model, serum irisin was independently and inversely associated with PDCI (OR = 0.685, 95% CI 0.500–0.939, *p =* 0.019). Age (OR = 1.082, 95% CI 1.043–1.122, *p <* 0.001), educational attainment (OR = 0.869, 95% CI 0.786–0.961, *p =* 0.006), and UPDRS‐III score (OR = 1.066, 95% CI 1.027–1.108, *p <* 0.001) were also independently associated with PDCI. Full statistical results are summarized in Table 4.

**TABLE 4:** Stepwise regression analysis of factors influencing cognitive impairment in PD Patients.

Feature	Analysis of factors			Multifactorial analysis		
	B	OR (95%CI)	*p* value	B	OR(95%CI)	P‐value
Age (years)	0.087	1.090 (1.058–1.124)	< 0.001	0.079	1.082 (1.043–1.122)	< 0.001
Level of education (years)	− 0.207	0.813 (0.744–0.888)	< 0.001	−0.140	0.869 (0.786–0.961)	0.006
Duration of illness (years)	0.248	1.281 (1.159–1.416)	< 0.001	0.057	1.059 (0.943‐1.189)	0.333
H‐Y stage	1.320	3.745 (2.507–5.593)	< 0.001	0.378	1.460 (0.757–2.815)	0.259
UPDRS III (score)	0.092	1.096 (1.067–1.125)	< 0.001	0.064	1.066 (1.027, 1.108)	< 0.001
Albumin (g/L)	− 0.077	0.926 (0.870–0.985)	0.015	− 0.048	1.050 (0.961–1.147)	0.283
Homocysteine (µmol/L)	0.092	1.096 (1.018–1.180)	0.016	0.031	1.032 (0.939–1.134)	0.514
Irisin (µg/mL)	− 0.269	0.764 (0.594–0.983)	0.036	− 0.378	0.685 (0.500–0.939)	0.019

To further explore the relationship between serum irisin levels and the severity of cognitive impairment, patients with PDCI were stratified into MoCA‐based milder and more severe cognitive‐impairment groups. Serum irisin levels did not differ significantly between the two groups [4.00 (3.12–4.52) vs. 3.55 (2.92–4.62) µg/mL, *p =* 0.559] (Table S1).

### 3.3 Correlation Between Serum Irisin Levels and Cognitive Domains

To further explore the association between serum irisin and cognitive function, PD patients were stratified into a low‐irisin group (*n =* 57, 23.5%) and a high‐irisin group (*n =* 186, 76.5%) based on the first quartile of irisin concentration. No significant differences were detected between the two groups in age or educational level (*p* > 0.05).

Patients in the low‐irisin group exhibited a higher prevalence of cognitive impairment compared with those in the high‐irisin group (68.4% vs. 50.0%, *p =* 0.015). Moreover, memory domain scores were significantly lower in the low‐irisin group [8.00 (7.00–10.00) vs. 9.50 (8.00–11.00), *p =* 0.010] (Figure 2A and Table 5). After adjusting for age and education, serum irisin levels were weakly but positively associated with memory performance, and this association remained significant after FDR correction across the five cognitive domains (*r =* 0.194, FDR‐adjusted *p =* 0.010) (Figure 2B and Table 6).

**FIGURE 2:**
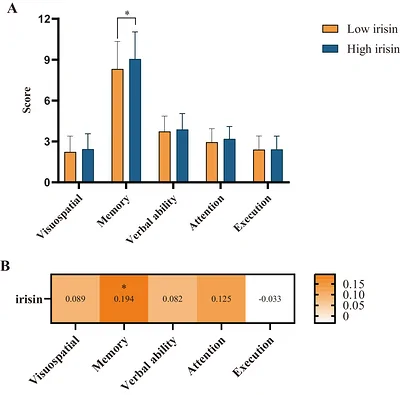
**(A)** Comparison of cognitive domain between low irisin and high irisin levels groups. The memory domain scores in the low irisin level group were significantly lower than those in the high irisin level group. (B) Heatmap of correlation analysis between serum irisin levels and cognitive domains. The darker the color in the graph, the stronger the correlation. The numbers on the graph represent the Spearman correlation coefficient. *: *p <* 0.05, **: *p <* 0.01.

**TABLE 5:** Comparison of general characteristics and cognitive domain between different serum irisin levels.

Feature	Low irisin (*n* = 57)	High irisin (*n* = 186)	z/t/χ2	P value
Age (years)	61.81 ± 10.60	61.58 ± 10.34	0.147	0.883
Level of education (years)	9.00 (5.00,9.00)	7.00 (5.00,9.00)	− 1.781	0.075
Duration of illness (years)	3.0 (2.0, 5.0)	3.0 (1.0, 6.0)	− 1.112	0.911
H‐Y stage	2.5 (1.5, 3.0)	2.0 (1.5, 3.0)	− 0.371	0.711
UPDRS III (score)	33.0 (20.5, 39.5)	29.0 (21.0, 43.2)	0.416	0.687
Cognitive impairment (n, %)	(39, 68.4%)	(93, 50.0%)	5.967	0.015
MMSE (score)	25.0 (23.0,26.0)	26.0 (24.0,28.0)	−3.048	0.002
MoCA (score)	20.0 (17.0,25.0)	24.5 (18.0,25.0)	−1.802	0.072
Visuospatial ability	2.00 (1.00,3.00)	3.00 (2.00,3.00)	−1.321	0.187
Memory	8.00 (7.00,10.00)	9.50 (8.00,11.00)	−2.562	0.010
Verbal ability	4.00 (3.00,5.00)	4.00 (3.00,5.00)	−1.120	0.263
Attention	3.00 (2.00,4.00)	3.00 (3.00,4.00)	−1.684	0.092
Execution	3.00 (2.00,3.00)	3.00 (2.00,3.00)	−0.065	0.948

**TABLE 6:** Analysis of the correlation between serum irisin levels and cognitive domains.

Cognitive domain	r	*p* value	FDR‐adjusted *p* value
Visuospatial ability	0.089	0.167	0.253
Memory	**0.194**	0.002^**^	0.010^*^
Verbal ability	0.082	0.202	0.253
Attention	0.125	0.052	0.130
Execution	−0.033	0.615	0.615

Abbreviations: R: Partial correlation coefficient.

**indicates *P* < 0.01, * indicates *P* < 0.05.

### 3.4 Association of Motor‐Subtype–Irisin Categories With Cognitive Impairment

To further explore the relationship between serum irisin levels, PD motor subtypes, and cognitive impairment, we categorized 93 patients with the TD subtype and 96 patients with the PIGD subtype into four groups according to both motor subtype and serum irisin level: TD–high irisin, TD–low irisin, PIGD–high irisin, and PIGD–low irisin.

After adjusting for age, educational level, and H–Y stage, multivariable logistic regression analysis demonstrated that the four‐level motor subtype–irisin categorical variable was associated with the presence of PDCI (overall *p =* 0.015). Compared with patients in the TD–high‐irisin group, those in the PIGD–low‐irisin group had higher odds of concurrent PDCI (OR = 6.065, 95% CI: 2.974–18.638, *p =* 0.002). Moreover, age (OR = 1.075, 95% CI: 1.034–1.118, *p <* 0.001), educational attainment (OR = 0.834, 95% CI: 0.736–0.944, *p <* 0.05), and UPDRS‐III score (OR = 1.077, 95% CI: 1.042–1.113, *p <* 0.001) were also independently associated with the presence of PDCI. Detailed statistical results are presented in Table 7.

**TABLE 7:** Analysis of factors influencing cognitive impairment in PD patients.

Feature	Analysis of factors			Multifactorial analysis		
	B	OR(95%CI)	*p* value	B	OR(95%CI)	*p* value
Age (years)	0.087	1.090 (1.058–1.124)	< 0.001	0.072	1.075 (1.034–1.118)	< 0.001
Level of education (years)	−0.207	0.813 (0.744–0.888)	< 0.001	−0.182	0.834 (0.736–0.944)	0.004
UPDRS III (score)	0.092	1.096 (1.067–1.125)	< 0.001	0.074	1.077 (1.042–1.113)	< 0.001
PD subtype‐irisin						0.015
TD‐high irisin	—	—	—		1 (reference value)	
TD‐low irisin	—	—	—	−0.063	0.939 (0.189–4.655)	0.939
PIGD‐high irisin	—	—	—	0.305	1.356 (0.571–3.222)	0.490
PIGD‐low irisin	—	—	—	1.803	6.065 (2.974–18.638)	0.002

Abbreviations: CI: confidence interval; OR: odds ratio; PIGD: postural instability and gait disorder; TD: tremor‐dominant.

## 4 Discussion

The main findings of this study are as follows: (1) cognitive impairment was common among patients with PD, particularly those with the PIGD subtype; (2) the PIGD subtype and lower serum irisin levels were associated with the presence of PDCI; and (3) among the four categories jointly defined by motor subtype and serum irisin level, patients in the PIGD‐low‐irisin group had higher odds of concurrent PDCI than those in the TD‐high‐irisin group.

In the present study, the prevalence of cognitive impairment in PD patients was 54.3%, which was significantly higher than that observed in healthy controls (17.8%), consistent with previous reports [24, 25]. Compared with patients with PDNCI, those with PDCI were older and had lower educational attainment—two factors well‐established associated with cognitive decline in PD [26‐28]. Furthermore, patients with PDCI exhibited longer disease duration, higher H–Y stage, and higher UPDRS III scores. After adjusting for age, educational level, and other confounding variables, UPDRS III score remained independently associated with the presence of PDCI,, suggesting that greater motor symptom severity was associated with concurrent cognitive impairment in this cohort.

Human evidence directly comparing peripheral irisin levels between patients with PD and healthy controls remains limited. A previous case‐control study reported modestly reduced plasma irisin levels in patients with PD (Shi et al. 2024), whereas our study found no significant difference in serum irisin levels between patients with PD and healthy controls. Differences in biofluid type, assay methodology, physical activity, muscle condition, and disease severity may partly account for these inconsistent findings. Intriguingly, stratified analyses within the PD cohort demonstrated that serum irisin levels were significantly lower in patients with PDCI than in those with PDNCI. Moreover, patients with the PIGD subtype had both a higher prevalence of cognitive impairment and lower serum irisin concentrations than those with the TD subtype. Lower serum irisin remained associated with the presence of PDCI after adjustment for potential confounders, consistent with the positive association between plasma irisin and MoCA scores reported by Shi et al. (2024). Taken together, this pattern suggests that variation in serum irisin within PD may be more closely related to clinical and functional heterogeneity than to the presence of PD itself.

We performed an exploratory subgroup analysis according to the severity of cognitive impairment. However, we did not observe a significant difference in serum irisin levels among subgroups with different degrees of cognitive impairment [4.00 (3.121, 4.52) vs. 3.55 (2.92, 4.62), *p =* 0.559]. This may be partly related to the limited sample size within each cognitive‐severity subgroup and the fact that cognitive impairment in the present study was defined using the MoCA rather than formal PD‐MCI or PDD diagnostic criteria.

Irisin, a bioactive peptide cleaved from FNDC5, is released in response to physical exercise and plays a pivotal role in regulating metabolism and glucose homeostasis. Accumulating evidence indicates that physical exercise mitigates cognitive decline through multiple pathways, with irisin serving as a key mediator of these neuroprotective effects (Zhang et al. 2022; Zhao et al. 2025; Zhao et al. 2025; Zhang et al. 2023; Islam et al. 2021; Xiao et al. 2025). In experiments using Alzheimer's disease (AD) mouse models, endurance exercise upregulated PGC‐1α expression, which subsequently enhanced FNDC5 expression, resulting in an approximately fourfold increase in BDNF levels and ultimately improving cognitive function in AD mice (Islam et al. 2021). Moreover, exogenously administered irisin has been shown to cross the blood–brain barrier, increase central irisin concentrations, and significantly ameliorate cognitive deficits and neuropathological alterations in AD mouse models (Lourenco et al. 2019; Pignataro et al. 2021). In a mouse model of vascular dementia (VaD), aerobic exercise increased endogenous irisin levels and promoted autophagy via the integrin αVβ5/AMPK/mTOR signaling pathway, while suppressing neuroinflammation and hippocampal neuronal apoptosis, thereby improving cognitive impairment induced by chronic cerebral hypoperfusion (Xiao et al. 2025). In addition, serum irisin levels increased after 12 weeks of regular exercise in patients with PD, and exogenous irisin improved motor function and attenuated dopaminergic neurodegeneration in animal models of PD (Zhang et al. 2023). Collectively, these findings provide biological plausibility for a relationship between irisin and cognition, but evidence from preclinical models cannot establish the mechanism underlying the associations observed in patients with PD. In the sample of this study, patients in the PIGD group had a longer disease duration, higher H‐Y grades, and gait and balance impairment that may restrict physical activity. Therefore, we hypothesize that as PD progresses, patients typically experience a decline in physical activity, particularly those with the PIGD subtype, who suffer from gait and balance impairments. Given that irisin secretion is closely linked to muscular activity, prolonged inactivity or restricted mobility may lead to decreased circulating irisin levels, thereby diminishing its neuroprotective influence. This reduction in irisin may partially explain why PIGD patients exhibit both lower irisin concentrations and higher rates of cognitive impairment. However, physical activity and muscle condition were not directly assessed in this study, and the cross‐sectional design cannot determine whether lower serum irisin reflects reduced mobility, is associated with cognitive impairment, or is related to other aspects of disease severity. Prospective longitudinal studies incorporating objective assessments of physical activity and muscle condition are needed to clarify these relationships.

Compared with other neurodegenerative disorders, PD is characterized by a more rapid and widespread decline across multiple cognitive domains—particularly in executive function, attention, visuospatial ability, and memory (Davis and Racette 2016). Among these, executive dysfunction and memory impairment are the most prominent (Das et al. 2016; Loftus et al. 2021). In the present study, patients with lower serum irisin levels exhibited significantly poorer performance in the memory domain compared with those with higher irisin levels. Moreover, after adjusting for age and education, serum irisin levels were weakly but positively associated with memory performance, and this association remained significant after FDR correction across the five cognitive domains (*r =* 0.194, FDR‐adjusted *p =* 0.010). This finding is biologically plausible in light of previous experimental evidence linking irisin to hippocampal‐dependent memory. For instance, in a streptozotocin‐induced diabetic animal model, irisin administration ameliorated memory and cognitive deficits (Wang et al. 2019). Similarly, Lourenco et al. reported that silencing FNDC5/irisin expression via short hairpin RNA in wild‐type mice impaired hippocampal synaptic plasticity and memory, whereas overexpressing FNDC5/irisin in AD model mice restored both (Lourenco et al. 2019). Nevertheless, the magnitude of the association in our cohort was weak, and the cross‐sectional data support only a modest association between serum irisin and memory performance. They do not establish a mechanistic or causal relationship in patients with PD. Its mechanistic role and clinical utility require further validation in longitudinal studies and mechanistic experiments.

Although the pathogenesis of cognitive impairment in PD remains incompletely understood, most studies have reported notable cognitive differences among patients with distinct motor subtypes. In 1990, Jankovic et al. (1990) proposed a comprehensive classification system for motor symptoms in PD and initially characterized the clinical features of each motor subtype. Their findings revealed significant cognitive disparities between TD and PIGD subtypes, with PIGD patients exhibiting a markedly higher prevalence and severity of cognitive impairment. These observations were subsequently confirmed by the prospective study of Alves et al. (2006) and the cohort study of van der Heeden et al. (2016). However, inconsistent results have also been reported—Lee et al. (2019) and Ren et al. (2020) found no significant cognitive differences among motor subtypes. In this study, patients with PD were classified into different motor subtypes based on the classic classification criteria for Parkinson's disease subtypes (Jankovic et al. 1990). The results showed that the prevalence of cognitive impairment was significantly higher in the PIGD group than in the TD group (68.8% vs. 46.2%), a finding consistent with the conclusions of most previous studies. The discrepancies among studies may be attributable to differences in classification criteria and heterogeneity in disease severity across study populations. Notably, both Lee et al. (2019) and Ren et al. (2020) recruited patients in the early stages of PD (disease duration < 1 year), which may explain their failure to detect cognitive differences. Previous studies have demonstrated a link between reduced gait speed and cognitive decline, with gait alterations often preceding measurable cognitive impairment (McGough et al. 2018; Kikkert et al. 2016). This relationship may be bidirectional, because cognitive dysfunction can adversely affect motor control, while gait and postural impairment may coexist with more extensive neurodegeneration. The PIGD phenotype and cognitive impairment may therefore reflect overlapping or parallel pathophysiological processes (Zuo et al. 2017).

The development of PDCI is multifactorial, and a single clinical or biochemical indicator is unlikely to fully capture its complexity. Therefore, combining motor subtype classification with serum irisin levels may provide additional information regarding the cognitive status of patients with PD. In the present study, patients with the PIGD subtype and low serum irisin levels showed higher odds of concurrent cognitive impairment than those with the TD subtype and high serum irisin levels (OR = 6.065, 95% CI: 2.974–18.638, *p =* 0.002). This finding provides a potential clinical perspective, suggesting that assessment of motor subtype together with serum irisin levels may contribute to a more comprehensive characterization of cognitive impairment in patients with PD. Although further longitudinal studies are needed to validate its clinical utility, this combined approach may help inform individualized follow‐up and management strategies.

Several limitations should be acknowledged. First, the cross‐sectional design precludes causal inference and prevents assessment of the temporal relationships among motor subtype, serum irisin, physical activity, and cognitive impairment. Second, physical activity was not quantified in this study. Therefore, we could not determine whether lower serum irisin levels were independently associated with cognitive impairment or partly reflected reduced mobility in patients with the PIGD subtype. Third, some healthy controls were recruited from spouses or siblings of patients with PD, which may have introduced shared lifestyle or genetic confounding. Finally, cognitive impairment and cognitive domain performance were assessed using the MoCA rather than a comprehensive neuropsychological battery or formal PD‐MCI/PDD diagnostic criteria. Although the MoCA is a useful screening tool, its domain‐specific subscores may not fully capture the complexity of cognitive dysfunction in PD, which may have limited the precision of cognitive domain classification and further subgroup analyses. Future large‐scale, multicenter longitudinal studies incorporating standardized physical activity assessment, formal cognitive diagnostic criteria, and repeated irisin measurements are needed to validate these associations and clarify the temporal and mechanistic relationships among motor subtype, irisin signaling, aging, and cognitive decline in PD. Moreover, mechanistic research exploring the interplay among aging, motor subtypes, and irisin signaling in PDCI pathogenesis is essential. Elucidating these mechanisms may lead to optimized intervention strategies and novel therapeutic targets, offering new hope for patients with PDCI.

## 5 Conclusions

Cognitive impairment was common among patients with PD, particularly those with the PIGD subtype. The PIGD subtype and lower serum irisin levels were associated with the presence of PDCI. In the joint‐category analysis, patients in the PIGD–low‐irisin group had higher odds of concurrent PDCI than those in the TD–high‐irisin group. Future prospective longitudinal studies are warranted to determine the temporal relationships among motor subtype, serum irisin levels, and cognitive impairment and to assess their associations with subsequent cognitive changes in patients with PD.

## Author Contributions

Meilin Gui designed the study, analyzed the data, and drafted the manuscript. Linlin Lv, Jinduo Huang, and Shenglan Hu designed the study, supervised and performed the procedure. Kexin Chen, Xing Wang, and Yiwen Zhou analyzed the data and prepared Figures 1–5. Meilin Gui, Linlin Lv., Beisha Tang, and Hainan Zhangrecruited patients and provided assessments, Chunyu Wang and Lixia Qin discussed and revised the manuscript. All authors reviewed the manuscript and approved the final manuscript before submission.

## Funding

This work was funded by the National Natural Science Foundation of China (82471277 to CW, 82101342 to LQ) and the National Science Foundation of Hunan Province (2024JJ5471).

## Ethics Statement

The study was conducted in accordance with the Declaration of Helsinki and approved by the Ethics Committee of the Second Xiangya Hospital of Central South University (LYEC2025‐0227).

## Consent

All participants in the study, as well as all authors of this article, signed written informed consent for the publication of this study.

## Conflicts of Interest

The authors declare no conflicts of interest.

## Supporting information




**Supplementary Material**: brb371848‐sup‐0001‐S1.docx

## Data Availability

Data that support the findings of this study are available from the first or corresponding author upon reasonable request and fulfillment of data sharing regulations approved by the local ethics committee. The data are not publicly available due to privacy or ethical restrictions.
