# Supplementary material for: Correlation Between Cognitive Function and Serum Irisin Levels in Parkinson's Disease Patients
Source: Brain Behav. 2026 Oct 21;16(10):e71848. doi: 10.1002/brb3.71848 (PMC13686351; doi:10.1002/brb3.71848)
Supplement: Supplementary file 1 [file BRB3-16-e71848-s001.docx]

Supplementary Table S1 Comparison of clinical characteristics according to MoCA-based cognitive-severity categories among patients with PDCI

| Feature | MoCA 21–25 (n=25) | MoCA <21（n=107） | F/H/χ2 | P value |
| --- | --- | --- | --- | --- |
| Sex female(n, %) | (9, 36.0%) | (59, 55.1%) | 2.972 | 0.085 |
| Age (years) | 66.24±10.449 | 65.07±9.143 | 0.558 | 0.578 |
| BMI(Kg/m^2^) | 24.42±3.30 | 23.24±3.37 | 1.579 | 0.117 |
| Level of education (years) | 3.00(1.00,5.00) | 5.00(3.00,8.00) | -2.530 | **0.011** |
| Irisin (μg/mL) | 4.00(3.121,4.52) | 3.55(2.92,4.62) | -0.584 | 0.559 |
| PIGD (n, %) | (8,32.0%） | (58, 54.2%） | 4.000 | 0.135 |
